# Effect of bed net colour and shape preferences on bed net usage: a secondary data analysis of the 2017 Malawi Malaria Indicator Survey

**DOI:** 10.1186/s12936-020-03499-9

**Published:** 2020-11-23

**Authors:** Donnie Mategula, Latif Ndeketa, Judy Gichuki, Boston Zimba, Wilson Ching’ani, Michael Give Chipeta

**Affiliations:** 1 Malawi-Liverpool Wellcome Trust Clinical Research Programme, Blantyre, Malawi; 2 Health Services Department, Nairobi City County Government, Nairobi, Kenya; 3 World Health Organization-Malawi Country Office, Lilongwe, Malawi; 4 Ministry of Health, Zomba District Health Office, Zomba, Malawi

**Keywords:** Malawi, Malaria, Bed net shape and colour, Bed net usage, Preferences

## Abstract

**Background:**

Malaria remains a significant cause of morbidity and mortality in the paediatric population in Malawi. Insecticide-treated bed nets are a key vector malaria control intervention, however, advancement towards universal access is progressing slowly. Malawi Malaria indicator surveys (MMIS) show diverse user preferences of bed net shape and colour. The objective of this work was to understand if bed net shape and colour preferences affect usage.

**Methods:**

This is a secondary analysis of data from households that participated in the 2016–2017 MMIS. The main outcome variable was net usage defined, at net level, whether someone slept under a particular net on the night before the survey. The main exposure variables were preference attributes, whether a particular net is of a preferred colour or shape as defined by the household respondent. Both bivariate and multivariate logistic regression analyses were done to determine the association between the exposure and outcome variables.

**Results:**

A total of 3729 households with 16,755 individuals were included in this analysis. There were a total 7710 bed nets in households that participated in the survey of which 5435 (70.5%) of these nets had someone sleep under them the previous night before the survey. Bed nets that are of a preferred shape have 3.55 times higher odds of being used than those not preferred [AOR 3.55 (95% CI 2.98, 4.23; *p* value < 0.001)]. Bed nets that are of a preferred colour have 1.61 times higher odds of being used than those that are not of a preferred colour [AOR 1.61 (95% CI 1.41, 1.84; *p* value < 0.001].

**Conclusions:**

The results indicate that if a bed net is of a preferred colour or shape, it is more likely to be used. Bed net purchase by malaria stakeholders need to balance more factors on top of preferences such as price and efficacy.

## Background

The burden of malaria disease in sub-Saharan Africa (SSA) remains a challenge. In 2017, out of the 219 million cases of malaria occurred worldwide, 90% were in SSA [1]. The predominant malaria parasite species within the region is *Plasmodium falciparum* and is responsible for a majority of the disease burden and mortality [2].

The World Health Organization (WHO) recommended a package of core interventions to ensure universal access to malaria prevention, diagnosis and treatment comprises vector control, chemoprevention, diagnostic testing and treatment [3]. At present, the two core, broadly-applicable vector control interventions are long-lasting insecticidal nets (LLIN) and indoor residual spraying (IRS) and it is within the WHO technical strategy to maximize the impact of these two interventions as progress towards malaria elimination is being made. Chemoprevention involves giving drugs to high-risk individuals, such as young children, pregnant women and non-immune travellers [4]. Also within the strategy, the WHO envisions that all suspected malaria cases are treated and that quality-assured treatment is available to all malaria patients [3].

Malaria remains a public health problem in Malawi. Main control measures include insecticide-treated bed nets and artemisinin-based combination therapy (ACT). Indoor residual spraying (IRS) has not been widely deployed and is currently used on a smaller scale in some parts of the country mainly due to the high costs of the non-resistant IRS compound [5–7]. Over the past decade, Malawi has substantially scaled up available malaria control tools, such as ITNs and ACT. During this period, the national parasite prevalence in children aged 2–10 has reduced by 47.2% (from 29.4–15.2% in 2010 and 2017, respectively) and mortality due to malaria has halved based on data available from the nationally representative surveys, such as the Malawi National Malaria Indicator Surveys (MMIS) that are conducted approximately every 2 years [8–10].

In 2017, the National Malaria Control Programme (NMCP) laid out a 5-year Malaria strategic plan (2018–2022). The strategy has two main aims; to reduce malaria incidence by at least 50% from a 2016 baseline of 386 per 1000 population to 193 per 1000 and reduce malaria deaths by at least 50% from 23 per 100,000 population to 12 per 100,000 population by 2022 [11]. These ambitious goals require increasing the intensity of the available control tools, such as bed net coverage and access to diagnosis and effective anti-malaria treatment.

The WHO recommends that universal access to and use of long-lasting insecticidal nets should be the goal for all people at risk of malaria. Access to an ITN is measured by the proportion of the population that could sleep under an ITN if each ITN in the household were used by up to two people. Comparing ITN access and ITN use indicators can help programmes identify if there is a behavioural gap in which available ITNs are not being used. The 2017 MMIS shows that the gap between access and use of ITN seems to have widened as compared to the 2014 and 2012 MMIS. Additionally, the access to use ratio has continued to decrease from 2012 (110.3%) to 2014 (87.9%) [12]. This is being observed while the control programme is making efforts through routine and mass net distribution to increase net access. This leaves the country to start looking at factors that could be affecting net usage.

Bed net user’s preference for certain net characteristics, e.g., mesh size, shape, colour, dimensions and fabric, may have a direct impact on net use [13]. In terms of malaria prevention, the concern arises when users do not use the publicly available nets or prefer purchased bed nets that may be untreated with insecticides, causing less protection against mosquito bites. Additionally, such preferences may influence the users to find alternative inappropriate practices, such as selling the nets [14] or using them for fishing [15].

In Malawi, bed nets obtained through the public sector either in routine or mass distribution are green in colour and rectangular shaped unlike those from the private sector that are predominantly blue or white and conical shaped. In assessing the colour of the nets during the 2017 MMIS, 88% of the observed nets were green, 7% light blue, 4% dark blue, and 1% white. However, when the household respondents were asked about bed net colour preference, 61% preferred the blue-, and 29% preferred the green- coloured bed nets. Additionally, in assessing the shape of the nets, 94% of the observed nets were rectangular and 6% were conical. However, 76% of respondents prefer conical, 21% prefer rectangular, and 2% did not have a clear preference. Notably, the trends for preferences in colour have changed from 38% green and 56% blue in the 2012 MIS to 29% green and 61% blue in the 2017 MIS and for preferences in shape have changed from 55% conical and 44% rectangular in the 2012 MIS to 76% conical and 21% rectangular in the 2017 MMIS. Published literature on the effect of user preferences suggests that factors that like bed net shape and colour preference do not significantly influence net use to degrees that would require action by programme planners [16]. Due to the widening gap of net access and use, in light of the diverse net preferences, analysis of the 2017 MMIS provides the latest evidence on the impact on net preferences and usage in Malawi.

## Materials and methods

### Study area

**Fig. 1:**
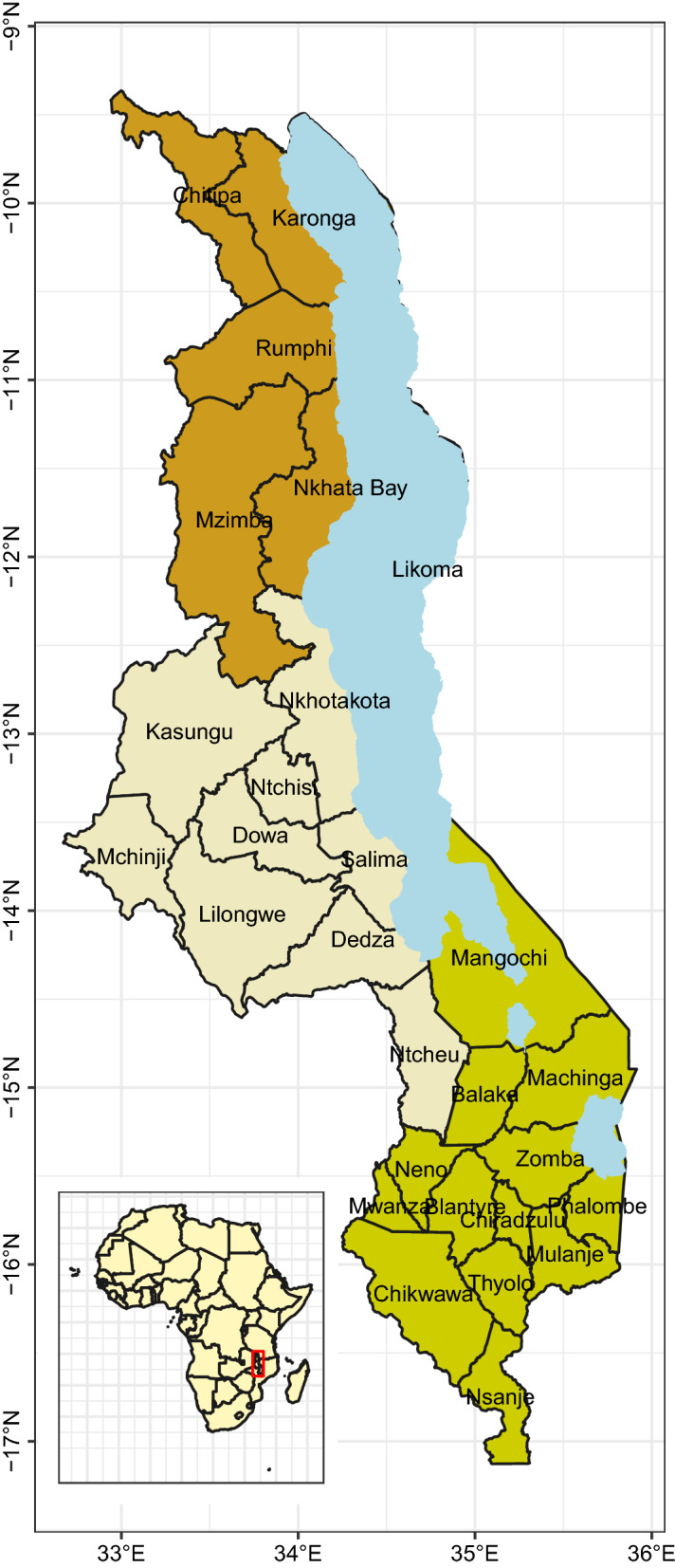
Three regions of Malawi, showing the 28 health districts

Malawi is a country located in the South Eastern region of Africa and is bordered by Tanzania, Mozambique and Zambia, see Fig. 1. Approximately 20% of the 118,484 km^2^ area is covered with water and the lowest point of the land lies at 37 m above sea level (MASL) while the highest point is at 3003 MASL. This makes the climate pattern in Malawi highly variable with temperatures averaging 14–32 °C [17] depending on altitude and proximity to the lake. Malawi has three seasons; hot–wet, hot–dry and cool–dry. From May to August, the weather is cool and dry, becomes hot in September and October, and the rainy season begins in October or November, continuing until April. The country is divided into three regions namely Northern, Central, and Southern regions. There are 28 districts in the country: 6 districts in the Northern region, 9 in the Central Region, and 13 in the Southern region (Fig. 1). The country has a population of 17.6 million [18] and a GDP per capita of 320 USD [19].

### Design and sample

The current secondary analysis uses cross-sectional household and household member and bed net data of most recent Malawi MIS conducted and published in 2017 [8]. The data used in this analysis were collected using the household questionnaire of the MMIS. The MMIS is a nationally representative survey conducted approximately every 2 years in Malawi and other low and lower-middle-income countries. In Malawi, MIS surveys have been done since 2004. These surveys are done to provide up-to-date estimates of demographic and health indicators related to malaria.

The 2017 MMIS followed a two-stage sampling design. In the first stage, 150 clusters were selected, with probability proportional to size using the Enumeration Areas (EAs) covered in the 2008 Malawi Population and Housing Census [20]. The second stage of sampling involved systematic sampling of 25 households in each of the EAs selected in the first stage. A total sample size of 3750 households was sampled. Those interviewed were all women age 15–49 years who were either permanent residents of the selected households or visitors who stayed in the household the night before the survey. Children aged between 6 and 59 months were tested for anaemia and malaria infection. During the MIS, of the 3750 households selected for the sample, 3735 were occupied at the time of fieldwork. Among the occupied households, 3729 were successfully interviewed and contained a total population of 16,755, providing the sample size used in the current analysis.

### Dependent study variable

Bed net usage was the outcome variable in the analyses and was measured whether or not someone slept in a particular net within the household on the night before the survey. In the MIS, this was collected through a questionnaire capturing household listing on individuals in that house and a net listing on that house. For every net in the house, respondents were asked who slept in the net the previous night. This data was combined and transformed to make the variable of individual-level net usage as a binary variable.

### Independent study variables

The current analysis aimed at assessing the association between bed net colour and shape preference to bed net usage, as such bed net colour preference and shape preference were the main explanatory variables. In the MIS, these variables were collected at household level through responses given by one survey respondent per household. To link these household-level bed net colour and shape preference data to the individual net usage data, a new binary variable designating whether a particular observed bed net is of the preferred colour (ispreferedcolour) or shape (ispreferedshape) was created. In this process, household data were linked to the net level data.

Bed net colour preference was originally in seven categories (blue, green, red, white, black, other and no preference), but it was re-categorized to four categories of blue, green, other and no preference to avoid data sparsity problems. Bed net shape preference was in three categories of rectangular, conical and no preference.

### Data management and statistical analysis

Household data and individual-level data were merged and cleaned, with some of the variables recoded to suit the objectives of the analyses. These processes were followed by exploratory and descriptive analyses to identify frequencies of household respondents, bed net usage, bed net shape and colour preferences and other background characteristics in relation to the outcome of bed net usage. In a bivariate analysis, the association of the two main exposure variables (net shape- and colour- preferences) and other background characteristics of the households to bed net usage defined above was explored using Pearson $\upchi$^2^ square tests but done and reported when logistic regression method was used. Crude odds ratios and their 95% confidence intervals were estimated. Likelihood ratio tests were then used to independently test the association of bed net colour and shape preference and bed net usage after adjusting for the background characteristics used in logistic regression models.

The two main explanatory variables namely colour and shape preferences were selected a priori after exploring the dataset. The rest of the variables were selected based on significance level, selecting all variables that were significant at a 5% significance level. Adjusted odds ratios with their 95% confidence intervals were estimated. The full logistic regression (model 1) used was compared with two reduced models (model 2) with main exposure variables and other significant covariates, model 3 with main exposure variables only, while checking the Akaike information criterion (AIC) and Bayesian information criterion (BIC) numbers to decide if indeed the full model was the best fitting model. All the analyses were done using R version 3.5.0 [21].

### Ethical consideration

Permission to use the dataset from was obtained from the Demographic and Health Surveys (DHS) Program, through the archiving office. The original study obtained ethical clearance from Malawi’s National Health Sciences Research Committee (NHSRC). All participants provided oral informed consent.

## Results

There were a total of 3729 households with a total population of 16,755 household members that participated in the survey. Table 1 shows the background characteristics of these households; a majority of which were in the rural areas N = 2239 (60%). The mean household size in the survey was 4.4 members (range 1–17, sd 2.1), with the mean number of rooms per household of 2.08 (sd 1.17). Detailed characteristics of these households are shown in Table 1.

**Table 1:** Characteristics of the households that participated in the 2017 MIS and bed net characteristics

Household (HH) characteristics			
		Residence	
	Total	Urban	Rural
Variable	n = 3729	n = 1490	n = 2239
Region			
North	1242 (100%)	499 (40.2%)	743 (59.8%)
Central	1243 (100%)	496 (39.9%)	747 (60.1%)
South	1244 (100%)	495 (39.8%)	749 (60.2%)
Household size	4.44 (2.08)	4.23 (2.03)	4.58 (2.11)
Number of rooms	2.08 (1.17)	2.19 (1.14)	2.00 (1.18)
Gender (HH head)			
Male	2822 (100%)	1136 (40.3%)	1686 (59.7%)
Female	907 (100%)	354 (39%)	553 (61%)
Age (HH head in years)			
15–25	1857 (100%)	800 (43.1%)	1057 (56.9%)
25–35	774 (100%)	355 (45.9%)	419 (54.1%)
35–45	505 (100%)	174 (34.5%)	331 (65.5%)
45 +	593 (100%)	161 (27.2%)	432 (72.8%)
Wealth			
Poorest	502 (100%)	0 (0%)	502 (100%)
Poorer	504 (100%)	19 (3.8%)	485 (96.2%)
Middle	523 (100%)	47 (9%)	476 (91%)
Richer	742 (100%)	214 (28.8%)	528 (71.2%)
Richest	1458 (100%)	1210 (83%)	248 (17%)
Net colour preference			
Blue	2234 (100%)	992 (44.4%)	1242 (55.6%)
Green	1064 (100%)	323 (30.4%)	741 (69.6%)
No preference	164 (100%)	48 (29.3%)	116 (70.7%)
Other	51 (100%)	10 (19.6%)	41 (80.4%)
White	216 (100%)	117 (54.2%)	99 (45.8%)
Net shape preference			
Conical	2856 (100%)	1311 (45.9%)	1545 (54.1%)
No preference	84 (100%)	22 (26.2%)	62 (73.8%)
Rectangular	789 (100%)	157 (19.9%)	632 (80.1%)
Bed net characteristics			
Net colour observed			
Blue	594 (100%)	367 (61.8%)	227 (38.2%)
Green	4746 (100%)	2035 (42.9%)	2711 (57.1%)
Other	19 (100%)	17 (89.5%)	2 (10.5%)
White	76 (100%)	63 (82.9%)	13 (17.1%)
Net has preferred colour			
No	3723 (100%)	1738 (46.7%)	1985 (53.3%)
Yes	1712 (100%)	744 (43.5%)	968 (56.5%)
Net shape observed			
Conical	561 (100%)	450 (80.2%)	111 (19.8%)
Other	6 (100%)	2 (33.3%)	4 (66.7%)
Rectangular	4868 (100%)	2030 (41.7%)	2838 (58.3%)
Net has preferred shape			
No	3981 (100%)	1859 (46.7%)	2122 (53.3%)
Yes	1454 (100%)	623 (42.8%)	831 (57.2%)
People per net			
1–2	4284 (100%)	2106 (49.2%)	2178 (50.8%)
3–4	1151 (100%)	376 (32.7%)	775 (67.3%)

**Fig. 2:**
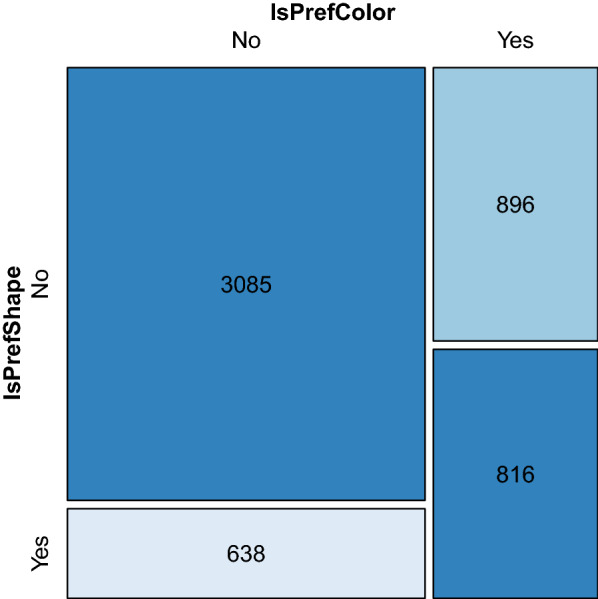
Cotabplot of preferred bed net colour and shape amongst those that used the bed net the previous night

There was a total of 7710 bed nets that were identified in households that participated in the survey of which 5435 of these nets had someone sleep under them the previous night before the survey, representing a bed net usage of 70.5%. The proportion of blue and green coloured nets used the previous night was 80.7% and 79.7, respectively (Chi^2^ P value = 0.058). The proportion of conical nets used the previous night was 83.4 while for rectangular nets was 79.7 (Chi^2^ P-value 0.0001). The most common observed colour is green with 4746 bed nets and the most common observed shape is rectangular with 4868 bed nets (See Additional File 1: Figure S1). Figure 2 shows whether people were using nets of their preferred colour and shape.

Table 2 shows the logistic regression crude and adjusted predictors of bed net usage. Results of the crude and adjusted analysis show that if a net is of a preferred colour or shape, there are higher odds that it will be used. Bed nets that are of a preferred shape have 3.55 times higher odd of being used than those not preferred (AOR 3.55 [95% CI 2.98, 4.23]; p-value < 0.001). Bed nets that are of a preferred colour have 1.61 times higher odds of being used than those that are not of a preferred colour (AOR 1.61 [95% CI 1.41, 1.84]; p-value < 0.001). In this modelling, ispreferedcolour, ispreferedshape, wealth index, the number of rooms in the household and the actual colour of the net are important predictors of the net usage (Additional Files 2, 3: Figs. S2, S3 ).

**Table 2:** Logistic regression crude and adjusted odds ratios of association between bed net shape and colour preference to usage

Logistic regression predicting bed net use: yes vs no					
	Crude OR (95% CI)	Crude P-value	Adj. OR (95% CI)	P (Wald’s test)	P (LR test)
Net shape					
Rectangular	Ref				< 0.001
Conical	0.21 (0.18–0.23)	< 0.001	0.16 (0.013–0.2)	< 0.001	
Other	0.07 (0.03–0.17)	< 0.001	0.09 (0.04–0.22)	< 0.001	
Net colour					
Green	Ref				< 0.001
Blue	0.18 (0.16–0.21)	< 0.001	0.19 (0.16–0.22)	< 0.001	
White	0.13 (0.1–0.17)	< 0.001	0.19 (0.14–0.27)	< 0.001	
Other	0.17 (0.1–0.17)	< 0.001	0.31 (0.16–0.58)	< 0.001	
Is preferred shape^a^					
No	Ref				< 0.001
Yes	1.02 (0.91–1.14)	0.702	3.55 (2.98–4.23)	< 0.001	
Is preferred colour^b^					
No	Ref				< 0.001
Yes	1.01 (0.91–1.13)	0.802	1.61 (1.41–1.84)	< 0.001	
Region					
Northern	Ref				< 0.001
Central	0.58 (0.52–0.66)	< 0.001	0.79 (0.69–0.91)	< 0.001	
South	1.03 (0.91–1.17)	0.612	1.35 (1.17–1.55)	0.001	
Wealth					
Lowest	Ref				< 0.001
Second	1.24 (1.02–1.51)	0.028	1.20 (0.96–1.51)	0.108	
Middle	1.63 (1.34–1.97)	< 0.001	1.46 (1.17–1.83)	< 0.001	
Fourth	1.71 (1.43–2.04)	< 0.001	1.60 (1.3–1.98)	< 0.001	
Highest	2.48 (2.11–2.91)	< 0.001	2.98 (2.45–3.63)	< 0.001	

^a^Whether or not the net colour that is preferred by the respondent of the household

^b^Whether or not the net shape that is preferred by the respondent of the household

**Fig. 3:**
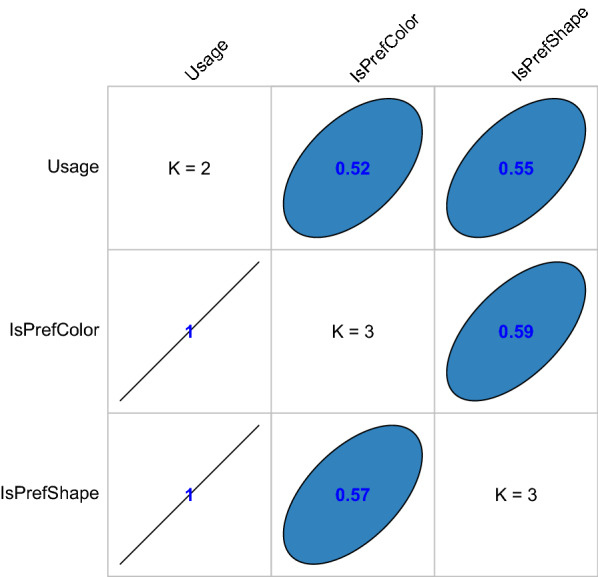
The association matrix for bed net usage and ispreferedcolour and isprefredshape

In Fig. 3, the association between bed net usage and the two main exposures, ispreferedcolur and ispreferedshape are shown. In both forward and backward association, these covariates are positively associated with bed net usage (forward association, ispreferedcolour 0.52 and isprefredshape 0.55; in backward association, both covariates have an association of 1).

Table 3 presents three logistic regression models predicting bed net usage. Model 1, bed net usage association with colour and shape preference adjusted for all available household covariates. Model 2, bed net usage association with colour and shape preference adjusting for all significant covariates only. Model 3, bed net usage association with colour and shape preference. Model 2 of all the three models has the least AIC and BIC values; indicating the least amount of information lost, hence the best model of the three and has been used to present main results shown in Table 2.

**Table 3:** Showing three different models

	Model 1	Model 2	Model 3
(Intercept)	1.98*** [1.48–2.65]	1.78*** [1.46–2.17]	3.34*** [3.11–3.58]
Net shape			
Rectangular	Ref		
Conical	0.16*** [0.13–0.20]	0.16*** [0.13–0.20]	0.19*** [0.16–0.23]
Other	0.09*** [0.03–0.22]	0.09*** [0.04–0.22]	0.07*** [0.03–0.19]
Net colour			
Green	Ref		
Blue	0.19*** [0.16–0.22]	0.19*** [0.16–0.22]	0.20*** [0.17–0.23]
White	0.19*** [0.14–0.27]	0.19*** [0.14–0.27]	0.22*** [0.16–0.30]
Other	0.30*** [0.16–0.58]	0.31*** [0.16–0.58]	0.30*** [0.16–0.55]
Is preferred shape			
No			
Yes	3.55*** [2.98–4.24]	3.55*** [2.98–4.23]	3.39*** [2.84–4.05]
Is preferred colour			
No	Ref		
Yes	1.61*** [1.41–1.84]	1.61*** [1.41–1.84]	1.61*** [1.41–1.83]
Region			
North	Ref		
Central	0.79*** [0.68–0.91]	0.79*** [0.69–0.91]	
South	1.34*** [1.17–1.55]	1.35*** [1.17–1.55]	
Number of rooms	1.17*** [1.08–1.27]	1.15*** [1.07–1.23]	
Wealth			
Poorest	Ref		
Poorer	1.19 [0.95–1.50]	1.20 [0.96–1.51]	
Middle	1.45** [1.16–1.82]	1.46*** [1.17–1.83]	
Richer	1.56*** [1.25–1.94]	1.60*** [1.30–1.98]	
Richest	2.78*** [2.17–3.56]	2.98*** [2.45–3.63]	
Residence			
Urban			
Rural	0.94 [0.79–1.12]		
People per net			
1–2			
3–4	0.98 [0.85–1.13]		
Household size	0.97 [0.91–1.04]		
Gender (HH head)			
Male	Ref		
Female	0.99 [0.87–1.14]		
Age(HH head in years)			
15–25	Ref		
25–35	0.98 [0.85–1.14]		
35–45	0.96 [0.81–1.14]		
45 +	0.92 [0.78–1.09]		
N	7710	7710	7710
AIC	7643.08	7631.47	7941.07
BIC	7795.99	7735.72	7996.68
Pseudo R2	0.29	0.29	0.24

Model 1, bed net usage association with colour and shape preference adjusted for all available household covariates. Model 2, bed net usage association with colour, adjusting for all significant covariates only. Model 3, bed net usage association with colour and shape preference

All continuous predictors are mean-centered and scaled by 1 standard deviation. ***p < 0.001; **p < 0.01; *p < 0.05

Table 3 shows three different models. Model 1, bed net usage association with colour and shape preference adjusted for all available household covariates. Model 2, bed net usage association with colour and shape preference adjusting for all significant covariates only. Model 3, bed net usage association with colour and shape preference.

## Discussion

Malawi remains a malaria-endemic country. The predicted *Plasmodium falciparum* parasite prevalence in the 2–10 years age group was 15.6% as of 2017 [7]. The current strategic plan of the country targets reducing malaria incidence and mortality by 50% from the 2015 baseline by 2022. To achieve these efforts, there is need to scale up current control strategies including the use of ITNs. A scale-up of ITN coverage and access is itself inadequate if strategies ensuring net usage are not in place.

The malaria surveys done in the country Malawi show diverse preferences for bed net shape and colour. The predominately preferred bed net is one that is blue coloured and conical shaped; the main reasons for this could be that they are easier to hang, they are a better fit around the sleeping space and that they are aesthetically more appealing [10, 22, 23]. The previous trends in preferences of bed net colour and shape from 2012 to 2017 [8, 10] inform the malaria community of a continued rate of change over time to the preference of bed nets that are blue and conically shaped. This is important for future procurement of bed nets for Malawi to maximize usage of distributed bed nets. The current bed nets that are distributed by the NMCP are rectangular and green shaped.

The present analysis shows that if a net is a preferred shape or colour as per household respondent, the odds of the net being used are higher. This finding is similar to what Koenker et al. found in their paper for Malawi in their analysis of their 2012 and 2014 MMIS data. Early qualitative studies done in Peru [13] suggested that user’s preferences on colour may impact usage. Pulford et al. in their systematic review, report several factors that contribute to non-use of bed nets including perceived discomfort with the net, technical factors such as inability to hang the net and travel [24]. In a recent similar secondary analysis of MIS data from Mali, Madagascar and Nigeria by Storey et al*.* bed net shape and colour preference were not included as some of the variables in the modelling, however, factors like age, household size, participation in net allocation were found to be predictors of net usage [25]. In the current analysis, it is shown that several factors are strong predictors of bed net usage. Some of which include the number of rooms in the house and the wealth index. The association of decreased net usage with increasing household size is also consistent with other studies. A study conducted in Nigeria found that for every increase in household size, the odds of bed net usage decrease by 13.8% (AOR 0.862 95% CI 0.822, 0.904; p-value < 0.001) [25]. A study conducted in Cameroon found that in houses where the net density is equal to or greater than 0.5, there were 8.8 higher odds of net usage compared to households where the density was less than 0.5 (AOR 8.88 95% CI 6.42, −12.64; p-value < 0.0001) [26].

In its efforts to scale up bed net usage, the Malawi National Malaria Control Programme (NMCP) routinely distributes bed nets to pregnant mothers and to under-five children. More bed nets are distributed to the wider population every 3–5 years through mass distribution campaigns. The type of bed nets used in these campaigns is mainly driven by cost. The current analysis informs the programme is consistent with the findings of Koenker et al*.* that the colour and shape of nets may impact usage. However, due to donor dependency on procuring and distribution of bed nets, the NMCP may not have full autonomy on the type of bed nets procured as such should also focus on other interventions that increase net usage. In a review by Augustincic et al. assessing interventions that increase bed net use, it was found that providing education of appropriate bet net use increases the number of adults and under-five children compared to giving no education. Providing incentives to encourage bed net usage has little or no benefit compared to no incentives. Distributing ITNs for free compared to making ITNs available for purchase through different mechanisms was also found to be effective in increasing net usage [27].

The findings of this study should be interpreted with several limitations and strengths in mind. First, the analyses used secondary data where some important independent variables that are known to influence bed net usage, such as participation in bed net allocation, were not available for inclusion in the logistic regression model. It is important to note that in the survey, preference of net colour and shape are household-level variables and individual nets were allocated to being preferred based on one household respondent. This should be interpreted with caution as the preferences of one individual do not necessarily represent the preferences of the entire household. It should also be noted that in this MIS, the majority of the nets used were the green coloured and rectangular shaped ones. As such the results may not be entirely generalizable. Further analysis would include adding person: net ratio and whether a net was purchased or not to the regression models. The strengths of this study include the use of nationally representative data with a large sample size, which imply robust statistical significance.

## Conclusion

It is known that blue is the preferred net colour whilst conical is the preferred net shape in Malawi. This paper shows that if a net is of a preferred colour or shape, it is more likely to be used. For malaria control stakeholders including funders, this result needs to be taken with caution as the choice of a bed net to procure needs to balance a lot more factors on top of preferences such as price and efficacy.

## Supplementary information

**Additional file 1**: **Figure S1**: The tabulation of observed bed net colour and shape. The most common observed colour is green with 4,746 bed nets and the most common observed shape is rectangular with 4,868 bed nets.

**Additional file 2**: **Figure S2**: The association matrix for bed net usage and covariates. Both forward and backward associations show that bed net usage is associated with observed bed colour, observed bed shape, the number of people per net, isprefcolour and isprefshape.

**Additional file 3**: **Figure S3**: The association between wealth an preference net shape. Chi square association p value of <  < 0.001.

## Abbreviations

AIC: Akaike information criterion
BIC: Bayesian information criterion
M/MIS: Malawi/malaria indicator survey
EA: Enumeration area
SSA: Sub-Saharan Africa
NMCP: National malaria control programme
A/OR: Adjusted/odds ratio
SD: Standard deviation
CI: Confidence interval
DHS: Demographic and Health Survey
NHSRC: National Health Sciences Research Committee
MASL: Metres above sea level
ITN: Insecticide-treated bed net
IRS: Indoor residual spraying
ACT: Artemisinin-based combination therapy
WHO: World Health Organization

## Availability of data and material

The datasets used and/or analyzed during the current study are available upon request from the Demographic Health Survey Programme Team. The request can be made via the link https://dhsprogram.com/Data/.
