# Supplementary figures and images for: Effect of bed net colour and shape preferences on bed net usage: a secondary data analysis of the 2017 Malawi Malaria Indicator Survey
Source: Malar J. 2020 Nov 23;19:428. doi: 10.1186/s12936-020-03499-9 (PMC7687838; doi:10.1186/s12936-020-03499-9)

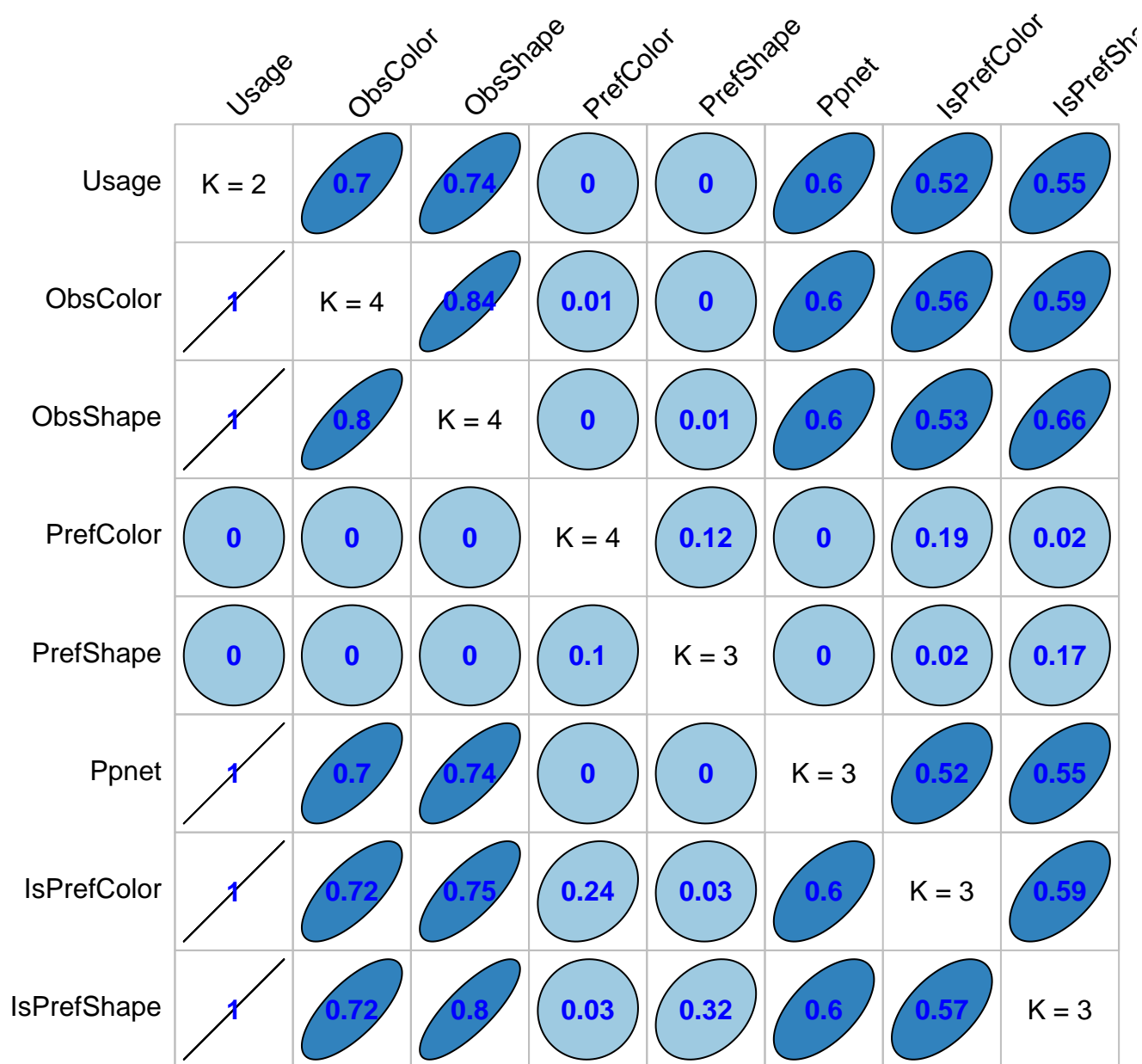

Supplement: Supplementary file 1 — Additional file 1: Figure S1: The tabulation of observed bed net colour and shape. The most common observed colour is green with 4,746 bed nets and the most common observed shape is rectangular with 4,868 bed nets. [file 12936_2020_3499_MOESM1_ESM.pdf]

PrefShape = Conical.

PrefShape = Rectang.

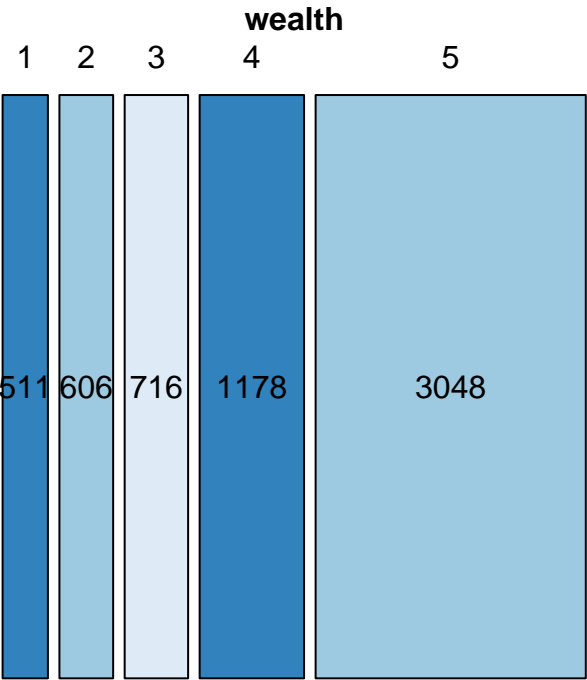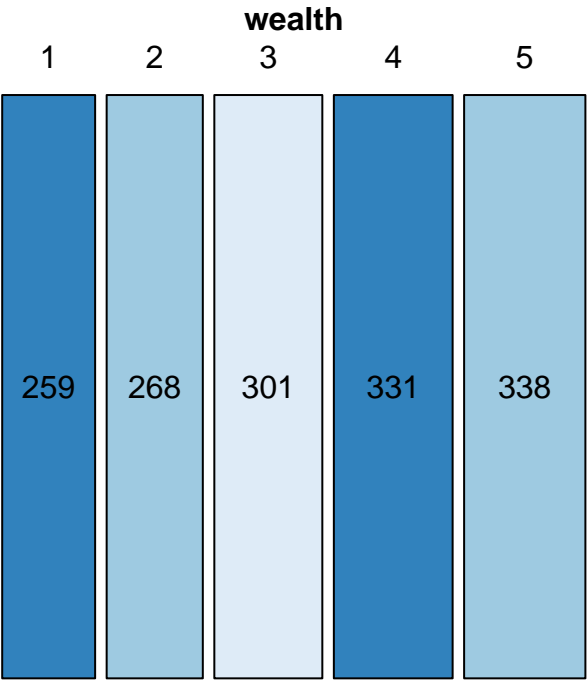

PrefShape = NoPref

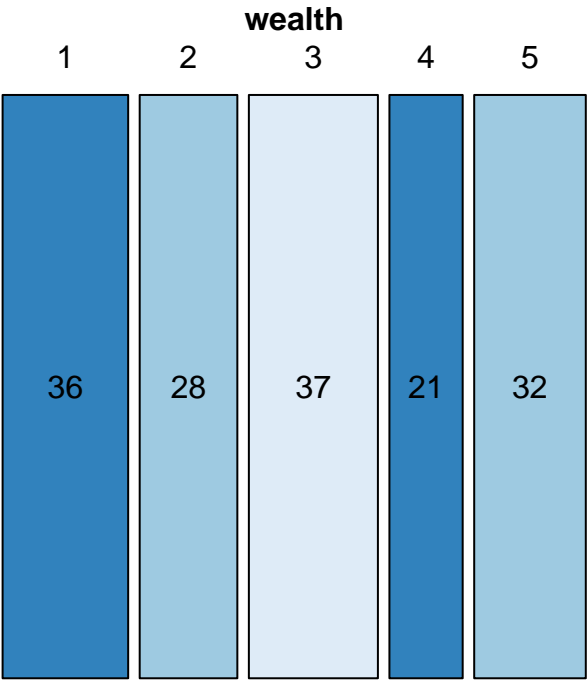

Supplement: Supplementary file 2 — Additional file 2: Figure S2: The association matrix for bed net usage and covariates. Both forward and backward associations show that bed net usage is associated with observed bed colour, observed bed shape, the number of people per net, isprefcolour and isprefshape. [file 12936_2020_3499_MOESM2_ESM.pdf]

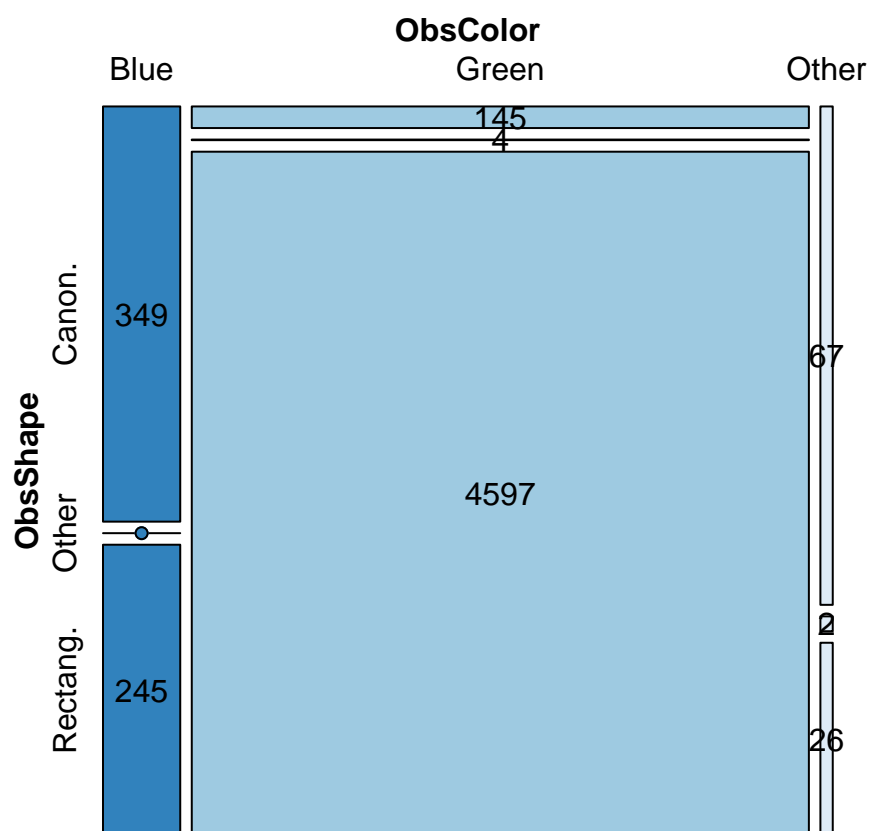

Supplement: Supplementary file 3 — Additional file 3: Figure S3: The association between wealth an preference net shape. Chi square association p value of < < 0.001. [file 12936_2020_3499_MOESM3_ESM.pdf]
